# Infant exposure to lithium through breast milk

**DOI:** 10.1192/j.eurpsy.2021.477

**Published:** 2021-08-13

**Authors:** M.L. Imaz, M. Torra, D. Soy, K. Langorh, L. Garcia-Esteve, R. Martin-Santos

**Affiliations:** 1 Unit Of Perinatal Mental Health Clinic-bcn, Department Of Psychiatry And Psychology, Institut Of Neuroscience, Institut D’investigacions Biomèdiques August Pi I Sunyer (idibaps), And Department Of Medicine, University Of Barcelona (ub), Hospital Clinic Barcelona, Barcelona, Spain; 2 Pharmacology And Toxicology Laboratory, Biochemistry And Molecular Genetics Service, Biomedical Diagnostic Center, Idibaps, Hospital Clinic Barcelona, Barcelona, Spain; 3 Division Of Medicines, Pharmacy Service, Idibaps, Hospital Clinic Barcelona, Barcelona, Spain; 4 Grass Research Group In Survival Analysis. Department Of Statistic And Operations Research., Universitat Politècnica de Catalunya, Barcelona, Spain; 5 Unit Of Perinatal Mental Health Clinic-bcn, Department Of Psychiatry And Psychology, Institut Of Neuroscience, Idibaps, Hospital Clinic Barcelona, Barcelona, Spain; 6 Psychiatry And Psychology Department, Centro De Investigación Biomédica En Red En Salud Mental (cibersam), Institut D’investigacions Biomèdiques August Pi I Sunyer (idibaps), Universitat Barcelona (ub), Hospital Clinic Barcelona, Barcelona, Spain

**Keywords:** lithium, Maternal breastfeeding, Formula feeding, Placental transfer

## Abstract

**Introduction:**

Women who take lithium during pregnancy and continue after delivery may opt to breastfeed, formula feed, or mix these options.

**Objectives:**

To evaluate the neonatal lithium plasma concentrations and nursing infant outcomes based on these three feeding trajectories.

**Methods:**

We followed 24 women with bipolar disorder on lithium monotherapy during late pregnancy and postpartum (8 per trajectory). Lithium serum concentrations were determined by an AVL 9180 electrolyte analyser with a 0.10 mEq/L detection limit and a 0.20 mEq/L limit of quantification (LoQ).

**Results:**

The mean ratio of lithium concentration in the umbilical cord to maternal serum being 1.12 (0.17). We used the Turnbull estimator for interval-censored data to estimate the probability that the LoQ was reached as a function of time. The median times to LoQ was 6–8, 7–8, and 53–60 days for formula, mixed, and breastfeeding, respectively. Generalised log-rank testing indicated that the median times to LoQ differed by feeding trajectory (p = 0.037). Multivariate analysis confirmed that the differences remained after adjusting for serum lithium concentrations at birth (formula, p = 0.015; mixed, p = 0.012). We did not found any acute observable growth or developmental delays in any of the neonates/infants.

**Conclusions:**

Lithium did not accumulate in the infant under either exclusive or mixed-breastfeeding. Lithium concentrations declined in all trayectories. The time needed to reach the LoQ was much longer for those breastfeeding exclusively. Lithium transfer via breastmilk is much less than via the placenta. We did not found any acute observable growth or developmental delays in any infant during follow-up.

**Disclosure:**

No significant relationships.

